# Sensation seeking as a potential screening tool for suicidality in adolescence

**DOI:** 10.1186/s12889-016-2729-2

**Published:** 2016-01-29

**Authors:** Won Kyung Lee, Dohee Lim, Hye Ah Lee, Hyesook Park

**Affiliations:** 1 Department of Social and Preventive Medicine, Inha University School of Medicine, 27 Inhang-Ro, Jung-Gu Incheon, Republic of Korea; 2 Department of Preventive Medicine, School of Medicine, Ewha Womans University, 1071 Anyangcheon-ro, Yangcheon-ku Seoul, 158-710 Republic of Korea

**Keywords:** Sensation seeking, Adolescence, Suicide, Depression

## Abstract

**Background:**

Although suicide could be an adverse health problem related to sensation seeking, this relationship has not been rigorously evaluated. The purpose of this study was to evaluate the relationship between sensation seeking and suicidality (suicidal ideation and plan) among adolescents and to test the influence of depressive symptom on this relationship.

**Methods:**

We surveyed 2,017 adolescents in seven middle and high schools located in urban and rural areas in 2012. A self-report questionnaire included items about demographic characteristics, sensation seeking, depressive symptom, and suicide plans. We evaluated the influence of sensation seeking on suicide plan using multiple logistic regression and causal mediation analysis.

**Results:**

Sensation seeking was related to suicide ideation and plan. Sensation seeking was associated with a 13 % greater likelihood of a suicide plan during the past 12 months as the score increased by 1. After controlling for depressive symptom, the effect of sensation seeking was reduced, but still significantly increased the risk (adjusted odds ratio: 1.10; 95 % CI: 1.04–1.16). When depressive symptom was included as a potential mediator, depressive symptom exerted an indirect effect on suicide planning that constituted 37 % of the total effect of sensation seeking. There was no significant interaction between sensation seeking and either demographic variables or depressive symptom.

**Conclusions:**

Sensation seeking can contribute to developing a suicide plan directly and indirectly via depressive symptom. Sensation seeking could be used to identify high-risk adolescents and provide proper interventions.

**Electronic supplementary material:**

The online version of this article (doi:10.1186/s12889-016-2729-2) contains supplementary material, which is available to authorized users.

## Background

Suicide is an important public health problem in adolescence. Globally, self-harm is related to 63,301 deaths each year and is the second leading cause of death among adolescents 15–19 years old. It has much greater influence than the general population whose suicide is the 14th greatest cause of death, accounting for 883,715 deaths in 2010 [1]. From the disease burden perspective, adolescents have been enduring heavier burden from self-harm than general population. Self-harm costs 4,373,730 disability-adjusted life years (DALYs) for ages 15–19, the 4th greatest contribution to total burden [2]. On contrast, it accounts for 11.9 % of 36,654,600 DALYs across all ages, ranked 18th in the general population. Although psychiatric disorders such as depression are often associated with suicide [3], there are insufficient screening tests for suicidality and the identification of high-risk individuals.

Sensation seeking is a personality trait characterized by “the willingness to take physical, social, legal and financial risks for the sake of seeking varied, novel, complex and intense sensation and experiences” [4]. Sensation seeking has been suggested to be related to risky driving, substance use, and risky sexual behavior [5]. Therefore, sensation seeking has been proposed to be a target for prevention programs for risk-related health problems including alcohol, drug usage, and sexually transmitted disease [6]. Suicide attempts may be an additional negative health behavior related to sensation seeking [7]. However, suicide is a mixed affective-behavioral psychopathology, and more research is needed to evaluate the relationship between sensation seeking and suicide and whether sensation seeking may be used as a screening test for suicidality [8]. Therefore, the purpose of this study was to evaluate the relationship between sensation seeking and suicidality (suicide ideation and plan) among adolescents. Additionally, we evaluated whether and how this relationship was influenced by depressive symptoms.

## Method

### Study design and participants

This study was a cross-sectional survey of students in middle and high schools located in urban, suburban, and rural areas (Seoul, Uijeongbu, and Nonsan) in Korea. We sampled a variety of seven feasible schools including girls’ and boys’ high schools and both standard- and advanced-curriculum schools. The survey population of seven schools was 5,439 which were all the students in seven schools and the sample population was 2,100 which came from 100 students per strata multiplied by 21 strata (3 grade X 7 schools). The classes were selected in a grade to survey at least 100 students and however the 3rd grade in high school was often impractical due to university admission schedule. The number of participants was 2,019 with overall response rate of 96.1 % and the enlisted students were slightly more female (*n* = 1,153) than male participants (*n* = 852). Approximately one-third of participants (*n* = 633) attended middle school and 1,375 attended high school. The median age of the participants was 16 years old and ranged from 13 to 18. The Ewha Womans University Institutional Review Board (IRB) approved this survey (IRB Number: 2012-07-18) and informed written consent was obtained from all participants. We explained the purpose and questionnaire of the study and got permission from the principals of the enrolled school. The school newsletter containing the survey plan was sent to the students’ families and parents had opportunity to withdraw consent. This process is similar to the Youth Health Study of Korea, which is a representative sample study on middle and high school students conducted by Korea Centers for Disease Control and Prevention to survey the health issues and risk factors [9].

### Measures

The questionnaire included items about demographic characteristics, sensation seeking, depressive symptom, and suicide (Additional file 1). Students answered questions regarding their experiences of suicidal ideation, planning, and attempts during the last 12 months. They also answered whether their experience of depressive symptoms during the last 12 months was enough to adversely affect their family and school life for a bout of 2 weeks or longer (“During the last 12 months, have you experienced trouble in doing normal day-to-day activities from sadness and loss of interest which persisted more than two weeks?”). Demographic characteristics included gender, grade, family income (low, lower-middle, middle, upper-middle and high) and number of siblings.

We used the Sensation-Seeking Scale developed by Slater (2003). It consists of two questions: “How often do you do dangerous things for fun?” and “How often do you do exciting things, even if they are dangerous?” rated using a 5-point scale from 1 (*not at all*) to 5 (*a lot*) [10]. This brief index of sensation seeking focuses on the risk-taking elements of sensation seeking and was developed for screening and large-scale surveys (Cronbach’s α = 0.81) [11]. This scale has very good internal validity and is correlated with substance use. We used sensation-seeking scale as a continuous variable in the logistic regression model and evaluated the odds ratio per a scale. For interpretation, the participants were divided into two group (high and low sensation seeker) in mediation analysis. High sensation seeking was defined as sensation seeking scores of 3.0 or greater, which corresponded to the 75th percentile in our study. Lower scores were considered indicative of low sensation seeking. The cutoff was chosen as the model using the 3rd quartile had lower AIC (Akaike Information Criterion) compared to those with the median, the 2nd tertile, and the 90th percentile [12–14].

### Data analysis

Demographic characteristics of gender, grade, family income, and number of siblings are presented in Table 1 based on extent of suicide ideation and planning. Multiple logistic regression was used to examine the effect of sensation seeking and demographic characteristics on suicide ideation and planning. The interactions between sensation seeking and both demographics and depressive symptoms were also evaluated. We evaluated the influence of sensation seeking on suicidality (suicidal ideation and plan) and whether this influence depended on one’s depressive symptoms. This was done by calculating depressive symptom as a mediator of an assumed causal relationship between sensation seeking and suicide planning. In this causal-mediation analysis, we included gender, grade, family income, and number of siblings as covariates. Analyses were conducted using R version 3.1.2 (R Foundation for Statistical Computing, Vienna, Austria) running the statistical package “mediation.” All reported *p*-values are two-sided and considered significant if less than 0.05.

**Table 1:** Participant demographics by suicide ideation and planning

	*N*	Suicide planning (*N* = 80)			Suicide ideation (*N* = 207)		
		*n*	%	*p*-value	*n*	%	*p*-value
Gender							
Male	852	37	4.3	0.495	90	10.6	0.769
Female	1,153	42	3.6		116	10.1	
Grade							
1st middle school	189	14	7.4	<0.01	32	16.9	<0.01
2nd middle school	250	16	6.4		43	17.5	
3rd middle school	194	10	5.2		22	11.4	
1st high school	607	18	3.0		50	8.3	
2nd high school	555	14	2.5		35	6.3	
3rd high school	213	7	3.3		24	11.3	
Family Income							
Low	80	7	8.8	<0.01	12	15.0	<0.01
Lower-middle	300	15	5.0		38	12.7	
Middle	1,064	28	2.6		80	7.5	
Upper-middle	430	24	5.6		59	13.8	
High	112	5	4.5		15	13.4	
No. of Siblings							
1	242	15	6.2	0.093	29	12.0	0.165
2	1,335	45	3.4		125	9.4	
≥ 3	418	19	4.5		51	12.2	
Sensation-seeking score^a^	4.0 (2.0–6.0)	5.0 (3.0–6.0)		<0.01	5.0 (3.0–6.0)		<0.01
Symptoms							
Non-depressive	1,683	31	1.8	<0.01	101	6.0	<0.01
Depressive	327	48	14.7		105	32.1	

Note: Percentages are based on different numbers of participants depending on frequency of answering

^a^Median and interquartile range (Q1-Q3), *P*-value for Wilcoxon rank sum test

## Results

### Suicidality and demographic variables

Two participants did not respond to the question regarding suicide ideation and plan during the past 12 months (response rate: 2,017 of 2,019 participants). Of these respondents, 10.3 % reported suicidal ideation, 4.0 % reported a suicide plan, and 2.1 % reported a suicide attempt. Suicide ideation and planning differed by grade, family income, sensation seeking, and depressive symptoms (Table 1). Specifically, middle school students reported more suicide planning than high school students. The percentage of students reporting suicide planning was lowest among students with middle- and high-income families. When it came to suicide ideation, the relationship to family income was likely to be U-shaped. Gender and number of siblings was not significantly related to suicide ideation and planning. More adolescents reported suicide planning in the high-sensation-seeking group. Total participants had the median value of 4 (interquartile range: 2–6), while those with suicidal ideation or plan had 5 (interquartile range: 3–6). Students with depressive symptoms were 26.1 and 12.9 %, respectively, more likely to report having suicidal idea and planned a suicide than non-depressed students.

### The influence of sensation seeking and depressive symptom on suicidality

The effects of sensation seeking and depressive symptoms on suicide ideation and planning are summarized in Table 2. As sensation seeking score increased by 1, students were 13 % more likely to have engaged in suicide planning. After controlling for demographics and depressive symptom, the risk of suicidal planning increased by 10 % as one sensation-seeking score rose. When it came to suicidal ideation and attempt, the adjusted odds ratios became 1.06 (95 % CI: 1.02–1.11) and 1.12 (95 % CI: 1.04–1.20), respectively.

**Table 2:** Effect of participant demographics and traits on suicide ideation and planning

	OR for suicide planning				OR for suicide ideation			
	COR	95 % CI	AOR^a^	95 % CI	COR	95 % CI	AOR^a^	95 % CI
Gender								
Male	1.09	0.89–1.33	1.02	0.80–1.30	1.03	0.89–1.19	0.96	0.81–1.14
Female	Ref		Ref		Ref		Ref	
Grade								
1st middle school	1.48	0.98–2.29	2.05	1.24–3.49	1.29	0.95–1.76	1.64	1.15–2.35
2nd middle school	1.37	0.92–2.09	1.76	1.10–2.94	1.30	0.98–1.74	1.57	1.13–2.19
3rd middle school	1.23	0.80–1.93	1.68	1.00–2.89	1.01	0.73–1.39	1.29	0.90–1.86
1st high school	0.96	0.66–1.43	1.16	0.75–1.88	0.84	0.45–1.10	0.97	0.82–1.31
2nd high school	0.89	0.61–1.34	1.11	0.71–1.81	0.73	0.55–0.96	0.92	0.60–1.12
3rd high school	Ref		Ref		Ref		Ref	
Family income								
1	1.79	1.14–2.68	1.67	1.01–2.64	1.49	1.04–2.11	1.40	0.94–2.04
2	1.34	1.00–1.78	1.30	0.93–1.80	1.35	1.09–1.66	1.35	1.06–1.70
3	Ref		Ref		Ref		Ref	
4	1.42	1.10–1.81	1.16	0.87–1.56	1.41	1.17–1.70	1.19	0.96–1.47
5	1.27	0.80–1.92	0.81	0.46–1.33	1.39	1.01–1.89	0.96	0.66–1.38
No. of siblings								
1	1.34	1.00–1.76	1.18	0.84–1.64			0.99	0.76–1.27
2	Ref		Ref		Ref		Ref	
≥3	1.15	0.89–1.46	1.11	0.83–1.46			1.15	0.94–1.40
Sensation seeking	1.13	1.08–1.19	1.10	1.04–1.16	1.10	1.06–1.14	1.06	1.02–1.11
Symptoms								
Non-depressive	Ref		Ref		Ref		Ref	
Depressive	2.82	2.27–3.51	2.85	2.25–3.62	2.97	2.51–3.52	3.00	2.51–3.60

^a^Adjusted for gender, grade, income, number of siblings, sensation seeking, and depressive symptom

When the participants were divided into high and low sensation seeker, those with sensation-seeking scores greater than the 3rd quartile had a 1.43 times (95 % CI: 1.12–1.81) greater likelihood of suicide planning than individuals scoring less than the 3rd quartile sensation-seeking. However, sensation seeking did not influence the relationship between depressive symptom and suicide planning. The odds ratio of depressive symptom on suicide planning only changed from 2.82 to 2.85 when demographics and sensation seeking were controlled in the model.

There were no significant interactions between demographic variables and sensation seeking or between depressive symptom and sensation seeking. Interactions between demographic variables and sensation seeking were not significant when the demographic variable was gender (*p*-value = .656), grade (*p*-value = .379), family income (*p*-value = .450) and sibling number (*p*-value = .531). When it came to the relationship between depressive symptom and sensation seeking, the significant interaction was not found (*p*-value = .338).

### The direct and indirect effects of sensation seeking and depressive symptom on suicidality

**Fig. 1:**
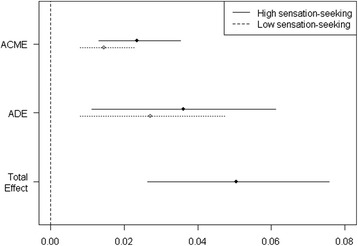
The mediated and direct effects of sensation seeking on suicide planning. ACME: average causal mediation effect (separately for high sensation-seeking and low sensation-seeking group). ADE: average direct effect (separately for high sensation-seeking and low sensation-seeking group). Total effect (Average)

The direct and indirect effects of sensation seeking on suicide planning were estimated using a model assuming that depressive symptom mediated this relationship (Fig. 1). In addition to a significant direct effect of sensation seeking on suicide planning, sensation seeking exerted an indirect effect via depressive symptom constituting 37 % of the total effect (95 % CI: 0.21–0.69). Figure 1 shows that high sensation seekers were more prone to suicide planning both due to the direct and indirect mediated effects, though the confidence intervals between high and low sensation seekers overlapped. Among low sensation seekers, the average mediated and direct effects on suicide planning were 0.014 and 0.027, respectively. In contrast, in the high-sensation-seeking group the corresponding values were 0.023 and 0.036, respectively.

## Discussion

This study was performed to evaluate the relationships between sensation seeking, depressive symptom, and suicide planning among adolescents. We found that sensation seeking was associated with increased suicide planning using a screening test for sensation seeking in a school-based survey. There were no significant interactions between demographic variables and sensation seeking or between depressive symptom and sensation seeking. Controlling for depressive symptom did not eliminate the relationship between sensation seeking and suicide planning.

Sensation seeking may contribute to suicide planning in several ways. Previous literature has suggested that sensation seeking has a unique influence on suicide ideation after controlling for other psychopathology [15]. Some researchers have included covariates to adjust for confounders such as depression and substance use [8]. Others have included depression and interpersonal dependency [16], and evaluated neuropsychiatric interviews and personality test [7, 17]. Additionally, a case–control study showed that the only difference between a suicidal and a non-suicidal group of the psychiatric outpatients with depression was novelty-seeking [18]. Other research has found that sensation seeking could be an independent contributor to suicidal ideation and attempting suicide [8, 19]. We found in this study that adolescents with high sensation seeking had a 43 % increased risk of developing a suicide plan (AOR: 1.43; 95 % CI: 1.12–1.81) controlling for demographic variables and depressive symptoms. This independent influence of sensation seeking can be considered consistent with the previous studies.

The independent influence of sensation seeking on suicide planning may be explained by several theories. One theory that supports the relationship between sensation seeking and suicide is the interpersonal-psychological theory of suicidal behavior [20, 21]. According to this perspective, two requirements of performing a suicide are a suicidal desire and the capability to act. The suicidal desire comes from one’s perceptions of burdening and social alienation. The capability to act develops through exposure and habituation to painful and fearsome experiences. Sensation seeking could also promoting one’s capability to act because it is associated with a fearlessness about death and an insensitivity to pain [22]. However, our results suggest that sensation seeking is not only related to suicide attempts but also suicidal ideation and planning. Therefore, another point of view may be helpful: suicidal thoughts and desires to pursue death could fall within the spectrum of risky behaviors that sensation-seeking individuals are more willing to do [23].

Our results also suggest that sensation seeking exerts an indirect influence on suicide planning. Suicidality is a multifaceted illness which has various interacting causes and risk factors [24]. Psychological traits and states could affect suicide planning in complex and interacting ways. One factor could be a positive or negative confounder to another factor, and mediate or moderate effects on suicide planning. Relationships may be difficult to reveal and nature of its relationship remains unclear. The relationship between sensation seeking and depressive symptom requires further investigation.

Sensation seeking has been considered to be the opposite of depressive symptom. The Cardiff Depression Study showed that sensation seeking was not associated with severe life events that are typically related to depression onset, and sensation seeking was negatively associated with depression [17]. Physiological evidence has suggested that individuals with bipolar affective disorder or sensation-seeking have increased evoked potential (especially the P2 slope), while unipolar affective disorders have decreased evoked potentials [25]. Other researchers have suggested that depression can be divided into two types (sub-threshold bipolar and true unipolar) by a hypomania checklist [26]. The sub-threshold bipolar group was more likely to exhibit sensation seeking, substance use disorder, and cyclothymic temperament.

In contrast, sensation seeking could be positively associated with depressive symptom. Zuckerman, who developed the sensation seeking scale, has said of sensation seeking that “it is assumed that the high sensation seeker will become bored and nonresponsive more quickly than other persons, when stimuli and experiences become repetitive” [16]. This suggests that sensation seekers could be more susceptible to feeling tiresome and bored for any given stimuli. One previous study showed that sensation seeking had a positive relationship to anhedonia which is a primary symptom of depression [27]. Another study was performed in the smokers before and during smoking cessation with a transdermal nicotine patch. Sensation seekers were more at risk of anhedonia, affective blunting, tiredness, and lack of energy [28]. However, little is known about this relationship and it still requires more research to clarify whether depressive symptom acts as a confounder or mediator. If depressive symptom acts as a mediator, the present study suggests that it contributes 37 % of the impact of sensation seeking on suicide planning.

This study has several limitations. First, the selection of schools may not be representative, although we covered seven middle and high schools located in urban, suburban, and rural areas. Second, data was collected using self-report questionnaires and some questions were not answered. Questions regarding family income had the lowest response rate at 98.4 %, while 99.9 and 99.6 % of students answered questions about suicide planning and depressive symptom, respectively. Third, to reduce participant burden, we used the short screening questionnaire for sensation seeking. Therefore, more detailed information on sensation seeking and other psychiatric problems was not available. Last, the causal assumptions of the mediation analysis could not be directly tested by our cross-sectional design. Therefore, interpretations regarding causes and mediations should be tentative. Moreover, study design was a cross-sectional survey and therefore further study is needed for causality.

## Conclusions

Our data suggest an influence of sensation seeking on suicide planning, independent of demographic variables and depressive symptoms. This study showed that a brief test for sensation seeking could contribute to effective screening for risk and susceptibility to suicide among adolescents. Our findings may be useful to developing school-based screening and intervention programs for high-risk sensation seekers. Education and interviews focused on this high-risk group could help to reduce rates of adolescent suicide.

## Additional file

Additional file 1:
**The questionnaire of youth injury-related behavior survey.** (DOCX 24 kb)
