# Supplementary material for: Sensation seeking as a potential screening tool for suicidality in adolescence
Source: BMC Public Health. 2016 Jan 29;16:92. doi: 10.1186/s12889-016-2729-2 (PMC4734849; doi:10.1186/s12889-016-2729-2)
Supplement: Additional file 1: — The questionnaire of youth injury-related behavior survey. (DOCX 24 kb) [file 12889_2016_2729_MOESM1_ESM.docx]

**Youth Injury-related Behavior Survey**

1. What is your sex?

① Male ② Female

2-1. What grade are you in?

① 1^st^ grade, middle school

② 2^nd^ grade, middle school

③ 3^rd^ grade, middle school

④ 1^st^ grade, high school

⑤ 2^nd^ grade, high school

⑥ 3^rd^ grade, high school

2-2. How old are you? ( ) years old

3-1. What is your birth order among your siblings?

( ) oldest among ( )

3-2. What is the number and gender of your siblings (excluding yourself)?

| The oldest | 1^st^ | 2^nd^ | 3^rd^ | 4^th^ | 5^th^ |
| --- | --- | --- | --- | --- | --- |
| Sex |  |  |  |  |  |

4. Who is in the family you are living with? (Select one or more responses.)

① Father ② Mother

③ Grandfather ④ Grandmother

⑤ Siblings ⑥ relatives ⑦ other

**The next 8 questions ask about intentional injuries.**

5. During the past 12 months, have you experienced violence by others?

① Yes ② No

6. During the past 12 months, have you experienced abuse by guardian?

① Yes ② No

7. During the past 12 months, have you experienced dating violence?

① Yes ② No

8. Up to now, have you experienced sexual assault?

① Yes ② No

9. During the last 12 months, have you experienced trouble in doing normal day-to-day activities from sadness and loss of interest which persisted more than two weeks?

① Yes ② No

10. During the past 12 months, did you ever seriously consider attempting suicide?

① Yes ② No

11. During the past 12 months, did you make a plan about how you would attempt suicide?

① Yes ② No

12. During the past 12 months, did you actually attempt suicide?

① Yes ② No

**The next 2 questions ask about safety.**

13. During the past 12 months, have you received safety-related education at school?

| Safety-related education | Yes | No |
| --- | --- | --- |
| 1) Traffic safety |  |  |
| 2) Kidnap/Disappearance |  |  |
| 3) Drug misuse and abuse |  |  |
| 4) Disaster |  |  |
| 5) Sexual assault |  |  |

14. How often do you do the following?

|  | Not at all | Rarely | Sometimes | Often | A lot |
| --- | --- | --- | --- | --- | --- |
| 1) How often do you do dangerous things for fun? | ① | ② | ③ | ④ | ⑤ |
| 2) How often do you do exciting things, even if they are dangerous? | ① | ② | ③ | ④ | ⑤ |

**The next 4 questions ask about unintentional injuries on school property.**

15. During the past 12 months, have you experienced injuries on school property?

① Yes ② No

16. During the past 12 months, have you experienced injuries in school playground?

① Yes ② No

17. During the past 12 months, have you been injured by being stuck on school property?

① Yes ② No

18. Up to now, have you experienced injured by falling on school property?

① Yes ② No

**The next 4 questions ask about road traffic injuries and related risk.**

19. During the last 7 days, how often did you jaywalk?

① 0/wk

② 1~2/wk

③ 3~4/wk

④ 5~6/wk

⑤ 1/d

⑥ 2/d

⑦ ≥3/d

20-21. During the last 7 days, how often did you use ( ) on the way home/school?

|  | 0/wk | 1~2/wk | 3~4/wk | 5~6/wk | 1/d |
| --- | --- | --- | --- | --- | --- |
| 20. bicycle | ① | ② | ③ | ④ | ⑤ |
| 21. school bus | ① | ② | ③ | ④ | ⑤ |

22. Do you have a driver license for motorcycle?

① Yes ② No

21-27. During the past 12 months, how often did you use ( )?

|  | 0/wk | 1~2/wk | 3~4/wk | 5~6/wk | 1/d |
| --- | --- | --- | --- | --- | --- |
| 21. Helmet while bicycling | ① | ② | ③ | ④ | ⑤ |
| 22. Helmet while driving a motorcycle | ① | ② | ③ | ④ | ⑤ |
| 23. Helmet while riding a motorcycle | ① | ② | ③ | ④ | ⑤ |
| 24. Protective equipment for sports | ① | ② | ③ | ④ | ⑤ |
| 25. Seatbelt in front seat of car | ① | ② | ③ | ④ | ⑤ |
| 26. Seatbelt in back seat of car | ① | ② | ③ | ④ | ⑤ |
| 27. Seatbelt in express bus | ① | ② | ③ | ④ | ⑤ |

**This is the end of the survey.**

**Thank you very much for your help.**
